# Validation of a risk prediction tool for coronary heart disease in middle-aged women

**DOI:** 10.1186/s12905-015-0250-x

**Published:** 2015-11-10

**Authors:** Katerina M. De Vito, Heather J. Baer, Hank Dart, Stephanie E. Chiuve, Eric B. Rimm, Graham A. Colditz

**Affiliations:** Division of General Internal Medicine and Primary Care, Brigham and Women’s Hospital, 1620 Tremont Street, Boston, Massachusetts 02120 USA; Department of Nutrition, Harvard T.H. Chan School of Public Health, Boston, Massachusetts USA; Department of Epidemiology, Harvard T.H. Chan School of Public Health, Boston, Massachusetts USA; Harvard Medical School, Boston, Massachusetts USA; Alvin J. Siteman Cancer Center and Department of Surgery, Washington University School of Medicine, St. Louis, Missouri USA; Division of Preventive Medicine, Brigham and Women’s Hospital, Boston, Massachusetts USA; Channing Division of Network Medicine, Brigham and Women’s Hospital, Boston, Massachusetts USA

**Keywords:** Coronary heart disease, Women, Risk assessment

## Abstract

**Background:**

Health risk appraisal tools may be useful for identifying individuals who would benefit from lifestyle changes and increased surveillance. We evaluated the validity of the Your Disease Risk tool (YDR) for estimating relative risk of coronary heart disease (CHD) among middle-aged women.

**Methods:**

We included 55,802 women in the Nurses’ Health Study who completed a mailed questionnaire about risk factors in 1994 and had no history of heart disease at that time. Participants were followed through 2004 for the occurrence of CHD. We estimated each woman’s 10-year relative risk of CHD using YDR, and we compared the estimated YDR relative risk category (ranging from “very much below average” to “very much above average”) to the observed relative risk for each category using logistic regression. We also examined the discriminatory accuracy of YDR using concordance statistics (c-statistics).

**Results:**

There were 1165 CHD events during the 10-year follow-up period. Compared to the “about average” category, the observed age-adjusted relative risk was 0.43 (95 % confidence interval: 0.33, 0.56) for the “very much below average” category and 2.48 (95 % confidence interval: 1.68, 3.67) for the “very much above average” category. The age-adjusted c-statistic for the model including the YDR relative risk category was 0.71 (95 % confidence interval: 0.69, 0.72). The model performed better in younger than older women.

**Conclusion:**

The YDR tool appears to have moderate validity for estimating 10-year relative risk of CHD in this population of middle-aged women. Further research should aim to improve the tool’s performance and to examine its validity in other populations.

**Electronic supplementary material:**

The online version of this article (doi:10.1186/s12905-015-0250-x) contains supplementary material, which is available to authorized users.

## Background

Coronary heart disease (CHD) is the leading cause of death in the United States, and rates continue to rise globally [1]. Many modifiable risk factors contribute to CHD incidence [2], and recent studies indicate that lifestyle factors may be the cause of the majority of CHD cases. The identification of individuals at high risk, who are most likely to benefit from lifestyle changes and increased surveillance, is important to decreasing morbidity and mortality from CHD.

Health risk appraisal tools may be useful for identifying individuals at increased risk and determining appropriate target populations for interventions [1–3]. Several risk prediction tools for CHD and cardiovascular disease (CVD) have been developed, such as the Framingham Risk Score and the PRECARD and PROCAM Risk Scores [4–13]. These tools, however, focus primarily on traditional clinical risk factors for CHD, utilize information that is not easily accessible to patients, and do not incorporate information on diet or physical activity, which are important risk factors for CHD [5–13].

Your Disease Risk (YDR) is a publicly-available, web-based tool (www.yourdiseaserisk.wustl.edu) that was designed to estimate risk of CHD and other chronic diseases [5]. Based on self-reported information on lifestyle and clinical risk factors, YDR estimates an individual’s relative risk of developing CHD over the next 10 years, compared to an average person of the same age and sex [5, 14]. Although the YDR risk assessment tool for CHD has been available on the Internet since 2004, it has not been formally evaluated. Given that this tool may be used by a large number of patients and clinicians for assessing and communicating about CHD risk, we feel that it is critical to examine its validity and to provide this information to the public. Therefore, the purpose of this study was to examine the validity of YDR for predicting relative risk of CHD among middle-aged women, using prospectively-collected data from the Nurses’ Health Study (NHS).

## Methods

### Study design and population

The NHS is a prospective cohort study that began in 1976, when 121,700 U.S. female registered nurses between ages 30 and 55 completed a mailed questionnaire about their lifestyle factors, health behaviors, and medical histories. Follow-up questionnaires have been sent to participants every 2 years since enrollment to update this information. Dietary factors were first assessed in 1980 and are updated every 4 years using a food frequency questionnaire [15]. The Institutional Review Board at Brigham and Women’s Hospital approved this study and considered completion of the questionnaires to be informed consent.

In this analysis, we included women who were alive in 1994 and completed the 1994 questionnaire. We chose 1994 through 2004 as the follow-up period for this analysis due to the availability of risk factor information as well as the age distribution of NHS participants; this allowed us to evaluate the performance of the YDR model in middle-aged and older women. We excluded women who had a history of myocardial infarction, coronary artery bypass surgery, or heart failure before 1994 (*n* = 37,085). We also excluded participants who missed 10 or more items on the 1994 food frequency questionnaire, reported eating less than 500 or more than 3500 kilocalories a day, or had missing data on any of the YDR risk factors (*n* = 9731). A total of 55,802 women were included in the final study population.

### Your Disease Risk Tool

The YDR tool for CHD was developed by a panel of epidemiologists, clinicians, and other experts from the medical community according to methods used to achieve consensus and risk classification for cancer over an extended period of time [5]. These methods were first used to develop the Harvard Cancer Risk Index, and are explained in detail in a report by Colditz et al [5]. The panel generated a list of genetic, environmental, nutritional, and lifestyle risk factors that are known causes of CHD or associated with CHD. Next, using the International Agency for Research on Cancer (IARC) classification approach, they classified the risk factors as “definite”, “probable”, and “possible” risk factors for CHD and assigned a magnitude of association for each factor [5, 16]. Prevalences for the risk factors in the U.S. population were estimated using many different sources, including data from the Centers for Disease Control’s Behavioral Risk Factor Surveillance System (BRFSS) [17] and the National Health and Nutrition Examination Survey (NHANES) [18]. Risk factors assessed in the YDR tool are body mass index (BMI) (for those <60 years old) and waist circumference (for those ≥60 years old), smoking history, exposure to secondhand smoke, dietary factors (collectively known as PrimeScreen [13], a brief 18-item diet assessment), use of vitamin supplements, physical activity, high blood pressure, diabetes, total cholesterol level, and family history of heart disease.

Each individual’s 10-year relative risk (RR) for developing CHD, compared to an average person of the same age and sex, is estimated using the following equation:

$$ RR\kern0.5em =\kern0.5em \frac{R{R}_{l1}*\kern0.5em R{R}_{l2}*\dots *R{R}_{1\mathrm{n}}}{\left[\left({P}_1*\kern0.5em R{R}_{C1}\right)+\left(1-{P}_1\right)*1.0\right]*\left[\left({P}_2*\kern0.5em R{R}_{C2}\right)+\left(1-{P}_2\right)*1.0\right]*\dots \left[\left({P}_n*\kern0.5em R{R}_{Cn}\right)+\left(1-{P}_n\right)*1.0\right]} $$

In this equation, RR_ln_ refers to the individual’s assigned relative risk (RR) for each risk factor, RR_Cn_ refers to the consensus-based relative risk for that risk factor, and P_n_ refers to the estimated U.S. population prevalence for that risk factor (See Table 1) [19]. In other words, the YDR equation uses data on each individual’s unique set of risk factors to estimate their relative risk of developing CHD over the next 10 years, compared to another person of the same age and sex in the general population. Each individual’s relative risk is estimated based on the consensus-based relative risk for each of their risk factors, as well as the prevalence of that risk factor in the U.S. general population. Based on the estimated YDR relative risk score calculated from this equation, each individual is then classified into one of seven relative risk categories, ranging from “very much below average risk” (estimated RR ≤ 0.2) to “very much above average risk” (estimated RR > 5.1) (5). For a sample calculation using the YDR equation, see Additional file 1.

**Table 1:** Comparison of observed prevalences of Your Disease Risk (YDR) risk factors with estimated U.S. population prevalences and observed relative risks of coronary heart disease with consensus–based relative risks among 55,802 Nurses’ Health Study (NHS) participants, 1994–2004

Risk factor	Observed prevalence of risk factor in NHS	Estimated U.S. population prevalence	Observed unadjusted RR(95 % CI) in NHS	Observed multivariate RR(95 % CI) in NHS ^a^	Consensus–based RR
	N (%)	%			
Overweight/Obesity					
BMI < 25 kg/m^2^ (Age < 60)	12,022 (44.3)	50.0	1.00 (REF)	1.00 (REF)	1.00 (REF)
BMI ≥ 25– <29 kg/m^2^ (Age < 60)	7480 (27.5)	20.0	1.21 (0.88–1.65)	1.02 (0.74–1.40)	2.25
BMI ≥ 29 kg/m^2^ (Age < 60)	7664 (28.2)	30.0	2.10 (1.60–2.75)	1.47 (1.09–1.97)	5.00
Waist ≤ 35in (Age ≥ 60)	24,381(85.1)	54.7	1.00 (REF)	1.00 (REF)	1.00 (REF)
Waist > 35in (Age ≥ 60)	4255 (14.9)	45.3	1.47 (1.24–1.74)	1.23 (1.03–1.46)	2.25
Smoking status					
Current smoker, ≥25 cig/day	1068 (1.9)	5.0	2.12 (1.50–2.98)	2.73 (1.92–3.89)	5.00
Current smoker, 15–24 cig/day	2552 (4.6)	10.0	2.48 (1.99–3.09)	3.15 (2.50–3.97)	2.25
Current smoker, ≤ 14 cig/day	2762 (5.0)	9.0	2.61 (2.12–3.21)	3.13 (2.51–3.89)	1.30
Past smoker, quit <2 years ago	1078 (1.9)	6.0	1.80 (1.25–2.60)	1.84 (1.27–2.67)	1.30
Past smoker, quit 2– < 10 years ago	5439 (9.8)	6.0	1.44 (1.18–1.76)	1.44 (1.17–1.76)	1.30
Past smoker, quit 10– < 20 years ago	5934 (10.6)	6.0	1.10 (0.89–1.36)	1.13 (0.91–1.40)	1.00
Past smoker, quit ≥20 years ago	11,673 (20.9)	10.0	1.11 (0.94–1.31)	1.09 (0.92–1.29)	1.00
Never smoked	25,296 (45.3)	48.0	1.00 (REF)	1.00 (REF)	1.00 (REF)
Exposure to secondhand smoke (past and never smokers)^b^					
Almost never or occasionally exposed	37,614 (67.4)	90.0	1.00 (REF)	1.00 (REF)	1.00 (REF)
Regularly exposed	18,188 (32.6)	10.0	1.03 (0.91–1.16)	1.20 (1.04–1.37)	1.30
Multivitamin or B complex					
≥5 times/week	23,497 (42.1)	60.0	1.01 (0.89–1.13)	1.02 (0.91–1.15)	0.80
<5 times/week	32,305 (57.9)	40.0	1.00 (REF)	1.00 (REF)	1.00 (REF)
Physical activity					
≥3 hours/week	11,870 (21.3)	19.0	0.79 (0.68–0.92)	0.85 (0.73–1.00)	0.55
<3 hours/week	43,932 (78.7)	81.0	1.00 (REF)	1.00 (REF)	1.00 (REF)
High blood pressure^c^					
Yes	14,227 (25.5)	27.5	2.05 (1.83–2.31)	1.51 (1.33–1.71)	2.25
No	41,575 (74.5)	72.5	1.00 (REF)	1.00 (REF)	1.00 (REF)
Diabetes or high blood sugar					
Yes	2180 (3.9)	8.1	4.41 (3.72–5.22)	3.20 (2.67–3.83)	2.25
No	53,622 (96.1)	91.9	1.00 (REF)	1.00 (REF)	1.00 (REF)
Total cholesterol level^d^					
≤159	3960(7.1)	14.0	1.00 (REF)	1.00 (REF)	1.00 (REF)
160–199	14,062 (25.2)	35.0	0.83 (0.63–1.09)	0.86 (0.66–1.13)	1.30
200–239	26,693 (47.8)	30.0	1.15 (0.90–1.47)	0.98 (0.76–1.26)	2.25
240–299	9542 (17.1)	15.0	1.48 (1.14–1.93)	1.26 (0.96–1.65)	5.00
≥300	1545 (2.8)	6.0	2.56 (1.83–3.58)	2.00 (1.43–2.82)	7.00
Family history of heart disease					
Yes	18,234 (32.7)	46.0	1.43 (1.27–1.60)	1.30 (1.15–1.46)	2.25
No	37,568 (67.3)	54.0	1.00 (REF)	1.00 (REF)	1.00 (REF)
Fish					
≥2 servings/week	13,961 (25.0)	35.0	0.91 (0.79–1.05)	0.90 (0.78–1.04)	0.55
<2 servings/week	41,841 (75.0)	65.0	1.00 (REF)	1.00 (REF)	1.00 (REF)
Fruits and vegetables					
≥5 servings/day	31,966 (57.3)	26.0	1.08 (0.96–1.21)	1.11 (0.98–1.26)	0.55
<5 servings/day	23,836 (42.7)	74.0	1.00 (REF)	1.00 (REF)	1.00 (REF)
Foods containing whole grains^e^					
≥3 servings/day	3605 (6.5)	11.0	0.83 (0.64–1.07)	0.79 (0.61–1.03)	0.55
<3 servings/day	52,197 (93.5)	89.0	1.00 (REF)	1.00 (REF)	1.00 (REF)
Nuts					
≥3 servings/week	13,077 (23.4)	12.0	1.06 (0.93–1.21)	0.99 (0.86–1.13)	0.80
<3 servings/week	42,725 (76.6)	88.0	1.00 (REF)	1.00 (REF)	1.00 (REF)
Foods containing saturated fat^f^					
≥2 servings/day	14,079 (25.2)	71.0	1.18 (1.03–1.34)	1.08 (0.95–1.24)	1.30
<2 servings/day	41,723 (74.8)	29.0	1.00 (REF)	1.00 (REF)	1.00 (REF)
Foods containing trans fat^g^					
≥5 servings/week	35,222 (63.1)	40.0	1.04 (0.92–1.18)	1.00 (0.89–1.14)	1.30
<5 servings/week	20,580 (36.9)	60.0	1.00 (REF)	1.00 (REF)	1.00 (REF)
Foods containing unsaturated^h^ fat					
≥5 servings/week	21,565 (38.7)	15.0	0.91 (0.80–1.02)	0.94 (0.83–1.06)	0.80
<5 servings/week	34,237 (61.4)	85.0	1.00 (REF)	1.00 (REF)	1.00 (REF)
Alcohol					
none	47,038 (84.3)	89.0	1.00 (REF)	1.00 (REF)	1.00 (REF)
≥1drink/day	8764 (15.7)	11.0	0.82 (0.69–0.97)	0.79 (0.66–0.94)	0.55

*REF* reference group, *RR* relative risk, *CI* confidence interval, *BMI* Body Mass Index

^a^Multivariate RRs are adjusted for all risk factors in table

^b^Observed prevalences and estimated US population prevalences do not include current smokers

^c^Those who indicated they were on high blood pressure medication were coded in the “yes” category

^d^Those who indicated “high cholesterol” without specifying a specific level and those who were on statins were coded in the “200–239” category

^e^Exposed group defined as those who ate 3or more servings a day of oats, cereal, cooked cereal, dark bread, brown rice, other grains, bran, popcorn, oat bran or wheat germ. YDR whole grain question text, “Do you eat 3 or more servings of whole grains per day (wheat bread, whole grain pasta, brown rice, oatmeal, whole grain breakfast cereal, bran or popcorn)?

^f^Exposed group defined as those who ate 2 or more servings a day of whole milk, butter, cream cheese, lard, red meat, cottage cheese, and other cheese. YDR saturated fat question text, “Do you usually eat butter, lard, red meat, cheese, or whole milk 2 or more times per day?”

^g^Exposed group defined as those who ate 5 or more servings per week of margarine, vegetable shortening, fried food, store–bought baked goods (pies, sweet rolls, cakes, cookies, donuts, or brownies). YDR trans fat question text, “Do you eat stick margarine, vegetable shortening, store–bought baked goods (cookies, cakes, pies) or deep–fried fast food on most days?”

^h^Exposed group defined as those who ate 5 or more servings per week of olive oil or vegetable oil. YDR unsaturated fat question text, “Do you eat oil–based salad dressing or use liquid vegetable oil for cooking on most days?”

### Outcomes

The outcome of interest was incident coronary heart disease (CHD), defined as fatal or nonfatal myocardial infarction (MI), occurring between return of the 1994 questionnaire and the end of follow-up on June 1, 2004. As previously stated, we chose 1994 through 2004 as the follow-up period for this analysis due to the availability of risk factor information as well as the age distribution of NHS participants. Nonfatal MI was confirmed using World Health Organization criteria along with a positive electrocardiogram test or elevated cardiac enzyme levels [15]. Data on deaths were collected via reports from next of kin, the postal service, and the National Death Index. Fatal MI cases were confirmed by patient medical records, autopsy reports, or death certificates [15].

### Assessment of risk factors

Information on risk factors in the study population was obtained from participants’ responses on the 1994 questionnaire or as close to 1994 as possible. Studies have validated self-reported data in the NHS [20–22]. Pearson correlations for measured versus self-reported values were 0.59 for physical activity, 0.94 for height, and 0.97 for weight; Pearson correlations for dietary factors ranged from 0.25–0.61 for intake of fruits, 0.30–0.47 for intake of vegetables, 0.58-0.79 for intake of whole grains, and was 0.41–0.59 for intake of fats (including saturated fat, trans fat and unsaturated fat) [20–22]. Previous studies also have demonstrated the validity of the shortened diet assessment, PrimeScreen [13], which is used in YDR. We re-coded all of the variables from the NHS data to be as consistent as possible with the risk factor definitions and categories that are used in the YDR tool (see Additional file 2 for details).

The YDR tool was specifically designed to use self-reported risk factor information at one point in time to estimate relative risk of developing CHD over the next 10 years. It does this based on self-reported information at a single point in time and does not update this information over time. Therefore, although updated risk factor information is available for participants in the NHS, we only used participants’ risk factor information as of 1994 for this analysis, in order to be consistent with how the YDR tool was intended to be used.

### Statistical analysis

We examined the observed prevalences of each risk factor in the NHS study population in 1994 and compared them to the estimated U.S. prevalences used in the YDR relative risk calculation. We then fit a series of logistic regression models with CHD as the outcome, first including each individual risk factor in a separate model and then including all of the individual risk factors in the same multivariate model, and compared the observed RRs for each risk factor in the study population to the consensus-based RRs used in the YDR relative risk calculation.

To examine the predictive ability of the YDR tool, we then fit a logistic regression model including indicator variables for the YDR relative risk categories as predictors of CHD. We assessed the calibration of the tool by comparing the observed RRs for each relative risk category in the study population to the estimated YDR relative risk category. To assess the discriminatory accuracy of the tool, we examined the area under the curve (AUC, or c-statistic) for the logistic regression model including indicator variables for the YDR relative risk categories and calculated 95 % confidence intervals for the c-statistics, overall and within 5-year age groups [23]. To evaluate the performance of the YDR tool in younger and older women, we used the Rosner and Glynn approach to compare the c-statistics of the model in women <60 years old and in women ≥ 60 years old [24].

We performed all statistical analyses using SAS version 9.3 (SAS Institute, Inc, Cary, NC).

## Results

The final study population for this analysis included 55,802 women, 97.9 % white, who were aged 47 to 74 in 1994. Women who were included were slightly younger than those who were excluded (mean age = 60.5 years versus 61.2 years). During the 10-year follow-up period, there were 1165 cases of incident CHD among women who were included in the study population (2.1 %). The 10-year risk of CHD was slightly higher among women who were excluded (3.3 %), which is not surprising given that one of main reasons for exclusion was a prior history of heart disease.

The observed prevalences of the YDR risk factors in the study population and the estimated U.S. population prevalences are shown in Table 1. The observed prevalences of some risk factors in the study population (e.g. physical activity, high blood pressure, and alcohol intake) were similar to the estimated U.S. prevalences, although others were somewhat different. For example, over 57 % of NHS study participants reported eating at least 5 servings of fruits and vegetables a day, compared to the estimated U.S. population prevalence of 26 %. Study participants also reported lower intake of foods containing saturated fat and higher intake of foods containing unsaturated fat and trans fat, compared to the population U.S. population estimates.

Table 1 also shows the observed RRs for each of the YDR risk factors in the study population and the consensus-based RRs. The observed RRs for some of the risk factors, such as obesity and smoking status, were weaker than the consensus-based RRs. However, most of the observed associations were in the expected direction and were fairly similar in magnitude to the consensus-based RRs.

The observed relative risks for CHD according to estimated YDR relative risk category are shown in Table 2. Although the observed RRs for each category were closer to 1 than the estimated RRs, the observed RRs did increase monotonically with increasing YDR category. Participants in the lower YDR relative risk categories had lower CHD risk compared to participants in the “average” YDR relative risk category; for example, the age-adjusted RR for participants in the “very much below average risk” category was 0.43 (95 % CI: 0.33, 0.56). Participants in the higher YDR relative risk categories had higher CHD risk compared to participants in the “average” YDR relative risk category; the age-adjusted RR for participants in the “very much above average risk” category was 2.48 (95 % CI: 1.68, 3.67). When we examined the mean age within each of the estimated YDR relative risk categories, we found that participants in the two highest YDR relative risk categories were slightly younger than participants in the other categories (data not shown), leading to confounding by age; this explains why adjusting for age led to changes in the observed relative risks. The results were similar when the analyses were stratified by age, although the observed RRs were slightly closer to the estimated YDR relative risk categories among participants who were younger than age 60 in 1994.

**Table 2:** Observed relative risks of coronary heart disease according to estimated Your Disease Risk (YDR) relative risk category among 55,802 Nurses’ Health Study participants, 1994–2004, overall and stratified by age in 1994

Estimated YDR relative risk category	*N* (%)	No. cases (total = 1165)	Observed RR (95 % CI)^a^	Age–adjusted RR(95 % CI)^b^
Very much below average (RR ≤ 0.2)	23,140 (41.5)	290	0.42 (0.32–0.55)	0.43 (0.33–0.56)
Much below average (0.2 < RR ≤ 0.5)	14,941 (26.8)	308	0.70 (0.53–0.91)	0.68 (0.52–0.89)
Below average (0.5 < RR ≤ 0.9)	7278 (13.0)	173	0.81 (0.61–1.07)	0.78 (0.59–1.04)
About average (0.9 < RR ≤ 1.1)	2321 (4.2)	68	1.00 (REF)	1.00 (REF)
Above average (1.1 < RR ≤ 2.1)	4369 (7.8)	166	1.31 (0.98–1.74)	1.32 (0.99–1.76)
Much above average (2.1 < RR ≤ 5.1)	2836 (5.1)	115	1.40 (1.03–1.90)	1.55 (1.14–2.11)
Very much above average (RR > 5.1)	917 (1.6)	45	1.71 (1.16–2.51)	2.48 (1.68–3.67)
Age < 60 (*N* = 27,166)				
Very much below average (RR ≤ 0.2)	11,390 (41.9)	56	0.39 (0.22–0.71)	0.40 (0.22–0.71)
Much below average (0.2 < RR ≤ 0.5)	6923 (25.5)	62	0.72 (0.40–1.29)	0.71 (0.40–1.28)
Below average (0.5 < RR ≤ 0.9)	3271 (12.0)	38	0.94 (0.51–1.73)	0.93 (0.50–1.72)
About average (0.9 < RR ≤ 1.1)	1129 (4.2)	14	1.00 (REF)	1.00 (REF)
Above average (1.1 < RR ≤ 2.1)	2156 (7.9)	47	1.78 (0.97–3.24)	1.73 (0.95–3.15)
Much above average (2.1 < RR ≤ 5.1)	1586 (5.8)	40	2.06 (1.12–3.81)	2.01 (1.09–3.72)
Very much above average (RR > 5.1)	711 (2.6)	26	3.02 (1.57–5.83)	2.97 (1.54–5.74)
Age ≥60 (*N* = 28,636)				
Very much below average (RR ≤ 0.2)	11,750 (41.0)	234	0.43 (0.32–0.58)	0.43 (0.32–0.59)
Much below average (0.2 < RR ≤ 0.5)	8018 (28.0)	246	0.67 (0.49–0.90)	0.67 (0.50–0.91)
Below average (0.5 < RR ≤ 0.9)	4007 (14.0)	135	0.74 (0.53–1.01)	0.74 (0.54–1.02)
About average (0.9 < RR ≤ 1.1)	1192 (4.2)	54	1.00 (REF)	1.00 (REF)
Above average (1.1 < RR ≤ 2.1)	2213 (7.7)	119	1.20 (0.86–1.67)	1.20 (0.87–1.67)
Much above average (2.1 < RR ≤ 5.1)	1250 (4.4)	75	1.35 (0.94–1.93)	1.40 (0.98–2.00)
Very much above average (RR > 5.1)	206 (0.7)	19	2.14 (1.24–3.69)	2.27 (1.32–3.93)

*REF* reference group, *RR* relative risk, *CI* confidence interval

^a^Logistic regression model includes indicator variables representing YDR relative risk categories

^b^Logistic regression model includes indicator variables representing YDR relative risk categories, and adjusts for age as a continuous variable

Table 3 displays the c-statistics for the logistic regression model with indicator variables for the YDR relative risk categories. The unadjusted c-statistic among all participants was 0.62 (95 % CI: 0.60–0.63), but this increased with adjustment for age; the age-adjusted c-statistic among all participants was 0.71 (95 % CI: 0.69, 0.72). When the analyses were stratified by age, the YDR relative risk category model had better discriminatory accuracy in younger age groups; the c-statistic was 0.71 (95 % CI: 0.69, 0.74) for those age 55 and younger, whereas it was 0.59 (95 % CI: 0.58, 0.61) for those over age 70. The c-statistics for the YDR relative risk category model among women who were less than age 60 and among women who were 60 or older were significantly different from one another (*p* = 0.001).

**Table 3:** Discriminatory accuracy for models with Your Disease Risk (YDR) relative risk category among 55,802 Nurses’ Health Study participants, 1994–2004, overall and stratified by age in 1994

	*N*	No. cases	YDR relative risk category model:^a^
C–statistic (95 % CI):			0.62 (0.60–0.63)
Age–adjusted c–statistic (95 % CI):			0.71 (0.69–0.72)
Age–specific c–statistic (95 % CI):			
≤55:	14,918	124	0.71 (0.69–0.74)
56–60:	12,469	160	0.66 (0.65–0.68)
61–65:	11,350	244	0.62 (0.61–0.64)
66–70:	11,222	386	0.62 (0.61–0.64)
>70:	5843	251	0.59 (0.58–0.61)

*RR* relative risk, *C-statistic* concordance statistic, *CI* confidence interval

^a^YDR relative risk category model contains indicator variables representing the YDR risk categories (Very much below average (RR ≤ 0.2), Much below average (0.2 < RR ≤ 0.5), Below average (0.5 < RR ≤ 0.9), About average (0.9 < RR ≤ 1.1), Above average (1.1 < RR ≤ 2.1), Much above average (2.1 < RR ≤ 5.1), Very much above average (RR > 5.1))

## Discussion

The purpose of this study was to evaluate the validity of the Your Disease Risk tool for estimating 10-year relative risk of CHD among middle-aged women. Since the YDR parameters were developed by a consensus-based process, this study represents an independent evaluation of the tool’s performance. Overall, our results show that YDR had moderate discriminatory accuracy in this population, as reflected by the unadjusted c-statistic, although the fit of the model improved substantially with adjustment for age. The YDR tool also seemed to perform better among the younger than the older women in this population.

Other validation studies of CHD risk prediction tools have had similar results as our study [6, 8, 9, 11, 12, 25]. For example, c-statistics for the Framingham model for predicting 5-year risk of CHD in an ethnically diverse population have ranged from 0.40 to 0.83 [23]. It is important to note that the Framingham model includes age and estimates absolute risk, while YDR does not include age and instead estimates an individual’s relative risk compared to someone of the same age and sex [5]. The discriminatory accuracy of the Framingham model was lower when evaluated in an older population (median age = 73), with c-statistics of 0.58 for both women and men [26]. These findings are fairly consistent with our results, since YDR also showed better discrimination in the younger than the older age groups. This may be because age is a more important risk factor for CHD in older individuals, whereas lifestyle factors may be more important in younger individuals and their role may lessen with age [25, 27–29].

In another recent paper that examined the validity of different cardiovascular risk prediction models in various populations, the original Framingham risk prediction algorithm for CHD had a c-statistic of 0.68 and the Adult Treatment Panel III risk prediction tool had a c-statistic of 0.71 in a multi-ethnic cohort of men and women aged 50–74 years [30]. Again, these values are similar to the c-statistics that were observed for the YDR tool in our study population. Viewed as a whole, these results indicate that the YDR tool – which uses only simple, self-reported risk factor information – has similar discriminatory ability as other CHD risk assessment tools that utilize more detailed clinical information.

Our study has several important strengths. The study population was large and was assembled from a well-established cohort with detailed information on many lifestyle and clinical risk factors. Our outcome of interest (CHD) was confirmed by medical records, rather than including self-reported diagnoses [15]. Unlike most other validated CHD risk prediction tools, YDR includes modifiable risk factors and only requires information that can be easily self-reported by patients (such as diet, physical activity, and general clinical risk factors). We calculated participants’ YDR relative risk scores as they would be calculated in a real-world setting; data from one point in time (1994 or as close to 1994 as possible) was used to calculate the YDR relative risk score over the next 10 years, and risk factor information was not updated over time. This is consistent with how the YDR tool was intended to be use.

Our study also has some limitations. We excluded a large number of women, which reduced the sample size and could affect the generalizability of our results. In addition, our study population included only nurses, who may be different than women in the general population. Although some studies have suggested that higher levels of education may be associated with healthier lifestyle choices and a more favorable cardiovascular risk factor profile [31], restriction of our study population to nurses does not imply that the results are only applicable to nurses. The YDR tool assigns a consensus-based relative risk to each risk factor that is included. In general, studies have shown similar associations between dietary and lifestyle factors and risk of chronic diseases, including CHD and cancer, in the NHS population and in other groups of women [32, 33]. Furthermore, the 10-year risk of CHD in our study population was 2.1 %, which is similar to other cohorts of women. For example, in a multi-racial cohort of middle-aged women in the Atherosclerosis Risk in Communities study, the observed risk of CHD was 1.9 % over 9.2 years of follow-up [34]. In a cohort of Danish women who were aged 30-63 at the beginning of the study, the 10-year risk of CHD was approximately 2 % [35]. These results show that the incidence of CHD in our cohort was fairly comparable to other groups of women.

In addition, the observed relative risks for the YDR categories generally were less strong than the relative risks estimated by the tool. This indicates that the YDR tool may not be well-calibrated in this population, especially in the extreme risk categories; it overestimated the risk in the lower categories, and underestimated the risk in the higher categories. In reality, the calibration of the YDR tool, as well as other risk prediction models, may depend on the distribution of risk factors in the population of interest.

Another limitation is that all of the risk factors were self-reported via mailed questionnaires, which could potentially lead to misclassification. However, several studies have shown that self-reported data on weight, height, waist circumference, physical activity, and dietary factors are valid in this cohort [20–22]; furthermore, YDR was designed specifically to utilize only information that can be easily reported by patients or the general public. Our findings do show that the observed RRs for some of the YDR risk factors in our study were different than the consensus-based RRs and different than in previous analyses in the NHS population. For example, the observed RR for CHD was 0.99 comparing intake of ≥ 3 servings/day of nuts vs. <3 servings/day, while a previous NHS analysis showed a strong inverse association for intake of nuts [36]. Similarly, the observed RR for CHD was 1.00 comparing those who consumed foods containing trans fat ≥5 times/week versus <5 times/week, whereas trans fat intake has been associated with elevated CHD risk in other NHS analyses [37]. There are several possible explanations for these differences. First, many of the variables are not assessed in the same way on the YDR tool and on the NHS questionnaires (see Additional file 2 for a side by side comparison of YDR and NHS variable categories). In particular, the questions about intake of certain foods that are used in the YDR tool are far less detailed than the food frequency questionnaires that are used to assess nutritional intake in the NHS. As a result, there is likely to be much more misclassification of dietary factors based on the YDR tool than in other NHS analyses, which would lead to attenuation of the observed associations. We also used information on dietary and other risk factors from only one point in time and did not update it over the course of the 10-year follow-up period, whereas other NHS analyses do update risk factor data over time; we performed the analysis in this manner to be consistent with how the YDR tool incorporates information on lifestyle and dietary factors. In addition, the observed RRs for the YDR risk factors in our study are for 10-year risk of CHD, while the consensus-based RRs refer to lifetime risk of CHD. Finally, with changing standards of care in the U.S., the impact of dietary and lifestyle factors on risk of CHD may have changed since the development of the YDR tool.

## Conclusion

In summary, the YDR tool appears to have moderate validity for estimating 10-year relative risk of CHD in this population of middle-aged women and to be comparable to other CHD risk prediction tools. Further research should aim to improve the performance of the tool and to examine its validity in other populations.

## Additional files

Additional file 1.
**“Sample YDR Relative Risk Calculation” shows a sample Your Disease Risk calculation for a hypothetical study participant.** (PDF 316 kb) Additional file 2.
**“Comparison of Your Disease Risk variable categories with NHS variable categories” is a table comparing Your Disease Risk variable categories and the Nurses’ Health Study variable categories side by side.** (PDF 188 kb)

## Abbreviations

YDR: Your Disease Risk
CHD: Coronary heart disease
NHS: Nurses’ Health Study
CVD: Cardiovascular disease
BRFSS: Behavioral Risk Factor Surveillance System
NHANES: National Health and Nutrition Examination Survey
RR: Relative Risk
P: Prevalence
MI: Myocardial infarction
AUC: Area under the curve
