# Supplementary material for: Validation of a risk prediction tool for coronary heart disease in middle-aged women
Source: BMC Womens Health. 2015 Nov 10;15:101. doi: 10.1186/s12905-015-0250-x (PMC4640388; doi:10.1186/s12905-015-0250-x)
Supplement: Additional file 1. — “Sample YDR Relative Risk Calculation” shows a sample Your Disease Risk calculation for a hypothetical study participant. (PDF 316 kb) [file 12905_2015_250_MOESM1_ESM.pdf]

Sample YDR Relative Risk Calculation

$$RR = \frac{RR_{I1} * RR_{I2} * \ldots * RR_{In}}{[(P_1 * RR_{C1}) + (1 - P_1) * 1.0] * \ldots [(P_n * RR_{Cn}) + (1 - P_n) * 1.0]}$$

RR_{In} =individual's assigned relative risk (RR) for each risk factor
R_{Cn} = consensus-based relative risk for that risk factor
P_n =consensus-based population prevalence of that risk factor

Consider a study participant with the following characteristics:
Female**,** 65 years old**,** overweight (waist circumference 40 inches), diabetic, smoker (30 cigs/day)
Plug in the consensus based RRs and consensus-based prevalences into the equation according to the woman's characteristics:

$$RR = \frac{RR_{I1} * RR_{I2} * \ldots * RR_{In}}{[(P_1 * RR_{C1}) + (1 - P_1) * 1.0] * \ldots [(P_n * RR_{Cn}) + (1 - P_n) * 1.0]}$$

$$RR = \frac{RR_{waist} * RR_{smk} * RR_{diab}}{[(P_{waist} * RR_{waist}) + (1 - P_{waist}) * 1.0] * \ldots [(P_{HDL} * RR_{HDL}) + (1 - P_{HDL}) * 1.0]}$$

$$RR = \frac{2.25 * 5.00 * 2.25}{[(0.453 * 2.25) + (1 - 0.453) * 1.0] * \ldots * [(0.125 * 2.25) + (1 - 0.125) * 1.0]}$$

$$RR = \frac{25.31}{10.24} = 2.47$$

YDR places this woman in the "much above average risk" for CHD compared to the average 65 year old woman.
