# Supplementary material for: Validation of a risk prediction tool for coronary heart disease in middle-aged women
Source: BMC Womens Health. 2015 Nov 10;15:101. doi: 10.1186/s12905-015-0250-x (PMC4640388; doi:10.1186/s12905-015-0250-x)
Supplement: Additional file 2. — “Comparison of Your Disease Risk variable categories with NHS variable categories” is a table comparing Your Disease Risk variable categories and the Nurses’ Health Study variable categories side by side. (PDF 188 kb) [file 12905_2015_250_MOESM2_ESM.pdf]

Comparison of Your Disease Risk variable categories with NHS variable categories

Variable	YDR question text	YDR risk factor categories	NHS variable categories
**Overweight/Obesity**	If <60: "What is your height?" & "What is your weight?"If ≥60 years old: "What is your waist circumference?"	BMI (if <60 yrs old)	1. Age<60, BMI< 25
		1. BMI<25	2. Age<60, BMI≥25 - <29
		2. BMI≥25-<29	3. Age<60, BMI≥ 29
		3. BMI≥ 29	4. Age>=60,Waist≤35
		Waist size (if ≥60 yrs old)	5. Age>=60,Waist>35
		1. Waist≤35in	
		2. Waist>35in	
**Smoking status**	"Do you smoke cigarettes?"If smoker: "How many cigarettes do you typically smoke per day?"If used to smoke: "How long ago did you quit smoking?"	1. Never smoked	1. Never smoked
		2. Current smoker, ≤ 14 cig/day	2. Current smoker, <14 cig/day
		3. Current smoker, 15-25 cig/day	3. Current smoker, 15-24 cig/day
		4. Current smoker, > 25 cig/day	4. Current smoker, ≥ 25 cig/day
		5. Past smoker, quit <2 years ago	5. Past smoker, quit <2 years ago
		6. Past smoker, quit 2-10 years ago	6. Past smoker, quit 2-10 years ago
		7. Past smoker, quit 10-20 years ago	7. Past smoker, quit 10-20 years ago
		8. Past smoker, quit ≥20 years ago	8. Past smokier, quit ≥20 years ago
**Second hand smoke exposure**	"How often are you exposed to second hand smoke?(Never or former smokers only)	1. Almost never/Occasionally exposed	1.Never and past smokers who are 'almost never or occasionally' exposed AND Current smokers
		2. Regularly exposed(*If never or former smoker)	2.Never and past smokers who are 'regularly' exposed
**Fish**	"Do you usually eat fish two or more times per week?"	1. Yes (≥2 serv/wk)	1. Yes (≥2 serv/wk)
		2. No (<2 serv/wk)	2. No (<2 serv/wk)
**Fruits and Vegetables**	"Do you eat 5 or more servings of fruit and vegetables per day?"	1. Yes (≥ 5 serv/day)	1. Yes (≥ 5 serv/day)
		2. No (<5 serv/day)	2. No (<5 serv/day)
**Foods containing**	"Do you eat 3 or	1. Yes (≥ 3 serv/day)	1. Yes (≥ 3 serv/day)

**whole grains**[a]	more servings of whole grains per day (wheat bread, whole grain pasta, brown rice, oatmeal, whole grain breakfast cereal, bran or popcorn?"	2. No (<3 serv/day)	2. No (<3 serv/day)
**Nuts**[b]	"Do you usually eat 3 servings of nuts per week?"	1. Yes (≥ 3 serv/wk)	1. Yes (≥ 3 serv/wk)
		2. No (<3 serv/wk)	2. No (<3 serv/wk)
**Foods containing saturated fat**[c]	"Do you usually eat butter, lard, red meat, cheese or whole milk 2 or more times per day?"	1. Yes (≥ 2 serv/day)	1. Yes (≥ 2 serv/day)
		2. No (< 2 serv/day)	2. No (< 2 serv/day)
**Eat trans fat?**[d]	"Do you eat stick margarine, vegetable shortening, store-bought baked goods (cookies, cakes, pies) or deep-fried fast foods on most days?"	1. Yes	1. Yes (≥ 5 serv/wk)
		2. No	2. No (<5 serv/wk)
**Foods containing unsaturated fat**[e]	"Do you eat oil-based salad dressing or use liquid vegetable oil for cooking on most days?"	1. Yes	1. Yes (≥ 5 serv/wk)
		2. No	2. No (<5 serv/wk)
**Alcohol**	"How many servings of alcohol do you have on a typical day?"	1. 0	1. none
		2. 1 or more	2. 1 or more drink/day
**Multivitamin or B complex**	"Do you take a multivitamin or B complex supplement on most days?"	1. Yes	1. Yes (≥ 5 times/wk)
		2. No	2. No (<5 times/wk)
**Physical Activity**	"Do you walk	1. Yes	1. ≥3 hours/wk

	(or do other moderate activity) for at least 30 minutes on most days, or at least 3 hours per week?”	2. No	2. <3hours/wk
**High blood pressure**	“Have you ever been told that you have high blood pressure (hypertension) or have you ever been given blood pressure medication?”	1. Yes	1. Yes, high blood pressure or on high blood pressure medication
		2. No	2. No (no high blood pressure AND not on any high blood pressure medication)
**Diabetes or high blood sugar**	“Have you ever been told that you have diabetes or a problem with high blood sugar?”	1. Yes	1. Yes
		2. No	2. No
**Total cholesterol level**	"What is your total cholesterol level?"	1. ≤ 159	1. ≤ 159
		2. 160-199	2. 160-199
		3. 200-239 and those who indicated high cholesterol without giving a specific number	3. 200-239 and those who indicated high cholesterol but did not know specific level and those on statins
		4. 240-279	4. 240-299
		5. ≥ 280	5. ≥ 300
**Family history of heart disease**	"Has anyone in your immediate family (mother, father, sister, brother) had a heart attack?"	1. Yes	1. Yes, mother and/or father has had an MI
		2. No	2. No, neither mother no father has had an MI

[a]Includes wheat germ, oat bran, popcorn, bran, brown rice, dark bread, oats, cooked cereal, and other grains
[b]Includes peanut butter, peanuts, and other nuts
[c]Includes whole milk, butter, cream cheese, cottage cheese, red meat, and other cheese
[d]Includes margarine, store bought fries, pie, sweet roll, cake, cookies, donuts, brownies, fried food at home, and fried food at home
[e]Includes light mayonnaise, mayonnaise, olive oil, and olive oil based salad dressing
